# Anterior ethmoid and frontal sinus drainage pathways: five patterns formed and defined by their bony walls

**DOI:** 10.1007/s00405-022-07517-1

**Published:** 2022-07-08

**Authors:** Toru Kikawada, Yasutomo Araki, Takuya Okamoto

**Affiliations:** 1 Nose Clinic Tokyo, Yaesuguchi Daiei Bldg., 1-3-1 Kyobashi, Chuo-ku, Tokyo, 104-0031 Japan; 2 Department of Otorhinolaryngology, Keio University, Tokyo, Japan; 3 Department of Otorhinolaryngology, University of Tokyo, Tokyo, Japan

**Keywords:** Frontal sinus, Anterior ethmoid, Drainage pathways, Endoscopic sinus surgery, Uncinate process, Ethmoidal bulla, Frontal recess anatomy

## Abstract

**Purpose:**

To perform endoscopic sinus surgery safely and effectively, surgeons need to visualize the complex anatomy of the anterior ethmoid and frontal sinus region. Because this anatomy is so variable and individualized, the foundation of understanding lies in identifying, following, and visualizing the drainage pathway patterns and anticipating possible variations.

**Methods:**

We studied 100 sides (50 cases: 22 male, 28 female, aged 12–86, average age 46.5 years, ± 19.5) using computed tomography (CT) and multiplanar reconstruction (MPR) to identify and classify the drainage pathways leading to the frontal sinus and anterior ethmoidal cells.

**Results:**

Analysis revealed five patterns of drainage pathways defined by their bony walls: between the uncinate process and the lamina papyracea [UP–LP]; between the uncinate process and the middle turbinate [UP–MT]; between the uncinate process and the accessory uncinate process [UP–UPa]; between the uncinate process and the basal lamella of the ethmoidal bulla [UP–BLEB]; and between the basal lamella of the ethmoidal bulla and the basal lamella of the middle turbinate [BLEB–BLMT]. In most cases, BLEB formed the posterior wall of the drainage pathway of the frontal sinus, indicating BLEB could be one of the most important landmarks for approaching the frontal sinus.

**Conclusions:**

As endoscopic sinus surgery depends on an understanding of this anatomy, this study may help surgeons to identify and follow the drainage pathways more accurately and safely through the anterior ethmoid to the frontal sinus.

## Introduction

The anatomy of the anterior ethmoid and frontal sinus is complex, but improvements in imaging technology are helping us to understand these spaces more clearly. Many recent reports have identified and classified the cells of the anterior ethmoid and frontal sinus based on their location [1–5]. However, the drainage pathways of the frontoethmoidal region are variable and their paths tortuous due to multiple bony walls which form the pathways.

In our study, the drainage pathways are formed by some combination of two of the following: the middle turbinate (MT), the basal lamella of the middle turbinate (BLMT), the uncinate process (UP) and its variations (UPa), the basal lamella of the ethmoidal bulla (BLEB), and the lamina papyracea (LP). We followed each drainage pathway to its termination as a cell (in the anterior ethmoid or frontal bone) or as a frontal sinus (in the frontal bone). The frontal sinus was determined based on location and size. Rather than focusing on cells, this study identifies the drainage pathways themselves to improve our understanding of the anatomy of the anterior ethmoid and frontal sinus.

## Materials and methods

Axial computed tomography (CT) scans were performed on Toshiba Alexion TSX-032A CT scanner using 1 mm thickness. InVesalius^©^ was used for three-dimensional (3D) and multiplanar reconstruction (MPR). CTs were obtained from June 2020 through October 2020 from 100 sides (50 cases: 22 male, 28 female, aged from 12 to 86, average age 46.5 years, ± 19.5). Exclusion criteria included previous sinus surgery or the presence of sinusitis. Inclusion required confirmation of drainage pathways to the frontoethmoidal region.

To begin identification of a drainage pathway, we started in the middle meatus and followed the drainage pathway formed by the bony walls of the anterior ethmoid to its termination, which might be the frontal sinus or an anterior ethmoidal cell or cells. Anterior ethmoidal cells included any frontal cells at the transition from ethmoidal to frontal bone, not including the frontal sinus. The word “cell” in this paper may refer to a pneumatized DP or a terminal recess; these are difficult to differentiate. We considered the frontal sinus to be the largest cell in contact with the squamous portion of the frontal bone.

In the “fronto-ethmoidal transition region,” coined by Zinreich et al., it can be difficult to distinguish between the uncinate process and the basal lamella of the ethmoidal bulla because of the labyrinthine nature of the ethmoidal complex [6]. We used MPR to trace continuity from the lower UP and BLEB to differentiate UP from BLEB. In some cases, an accessory lamella (UPa) forms from UP or BLEB. We counted only the frontal sinus, not other cells occurring in the frontoethmoidal region.

## Results

After we had identified and named the five drainage pathway patterns, we calculated the prevalence for each pattern with 95% confidence interval (CI). Table 1 shows the prevalence of each drainage pathway pattern, the prevalence of each pattern terminating in a frontal sinus, and the prevalence of frontal sinus formation within each drainage pathway.

**Table 1:** Prevalence of drainage pathways leading to frontal sinus

Drainage Pathway (DP)	[DP]/sides		[DP- > FS]/sides		[DP- > FS]/DP	
	Prevalence	95% CI	Prevalence	95% CI	Prevalence	95% CI
[UP–LP]	100% (100/100)	95.6–100.7	11% (11/100)	6.1–18.8	11% (11/100)	6.1–18.8
[UP–MT]	100% (100/100)	95.6–100.7	46% (46/100)	36.6–55.7	46% (46/100)	36.6–55.7
[UP–UPa]	18% (18/100)	11.6–26.7	14% (14/100)	8.4–22.3	77.8% (14/18)	54.3–91.5
[UP–BLEB]	64% (64/100)	54.2–72.7	24% (24/100)	16.6–33.3	37.5% (24/64)	26.6–49.8
[BLEB–BLMT]	100% (100/100)	95.6–100.7	5% (5/100)	1.9–11.5	5% (5/100)	1.9–11.5

*FS* frontal sinus, *UP* uncinate process, *LP* lamina papyracea, *MT* middle turbinate, *UPa* accessory uncinate process, *BLEB* basal lamella of ethmoidal bulla, *BLMT* basal lamella of middle turbinate

Note: For the figures, we selected 5 sides out of 100 total sides, both right and left. However, to reduce confusion and improve consistency, we inverted any left sides so that all figures appear as right sides.

### Notation of drainage pathways

For example:

[UP–LP] denotes the drainage pathway.

[UP–LP- > FS] denotes the drainage pathway leading to a frontal sinus.

[UP–LP- > C] denotes the drainage pathway leading to a cell or cells.

### The five drainage pathways of the anterior ethmoid and frontal sinus

**Fig. 1:**
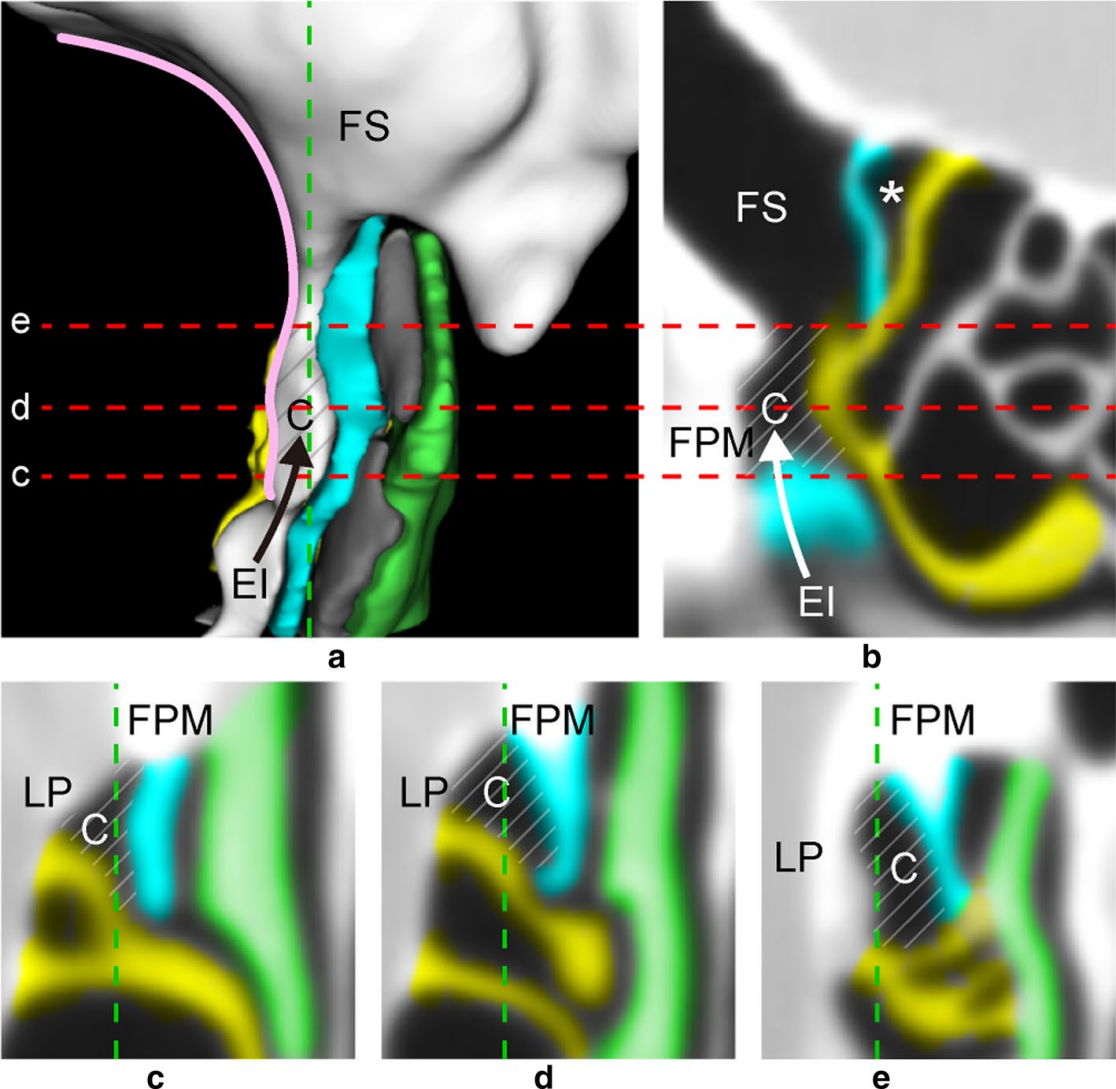
**a** Anterior view of 3D reconstruction of [UP–LP- > FS] arising from [UP–LP- > C] along LP (pink) from EI (black arrow). Grey indicates [UP–MT]. Vertical dotted line indicates sagittal slice (**b**). Horizontal dotted lines indicate axial slices (**c**–**e**). **b** Sagittal MPR. Note [UP–LP- > C] arises from EI (white arrow) then extends superiorly along FPM to form [UP–LP- > FS]. The anterior margin of BLEB connects to the skull base, forming the posterior wall of [UP–LP- > FS] and, in this case, [UP–BLEB- > C] (*). **c**–**e** Axial MPR. blue = uncinate process (UP); yellow = basal lamella of ethmoidal bulla (BLEB); green = middle turbinate (MT); diagonal lines = [UP–LP]; *EI* ethmoidal infundibulum, *C* cell, *FS* frontal sinus, *LP* lamina papyracea

**Fig. 2:**
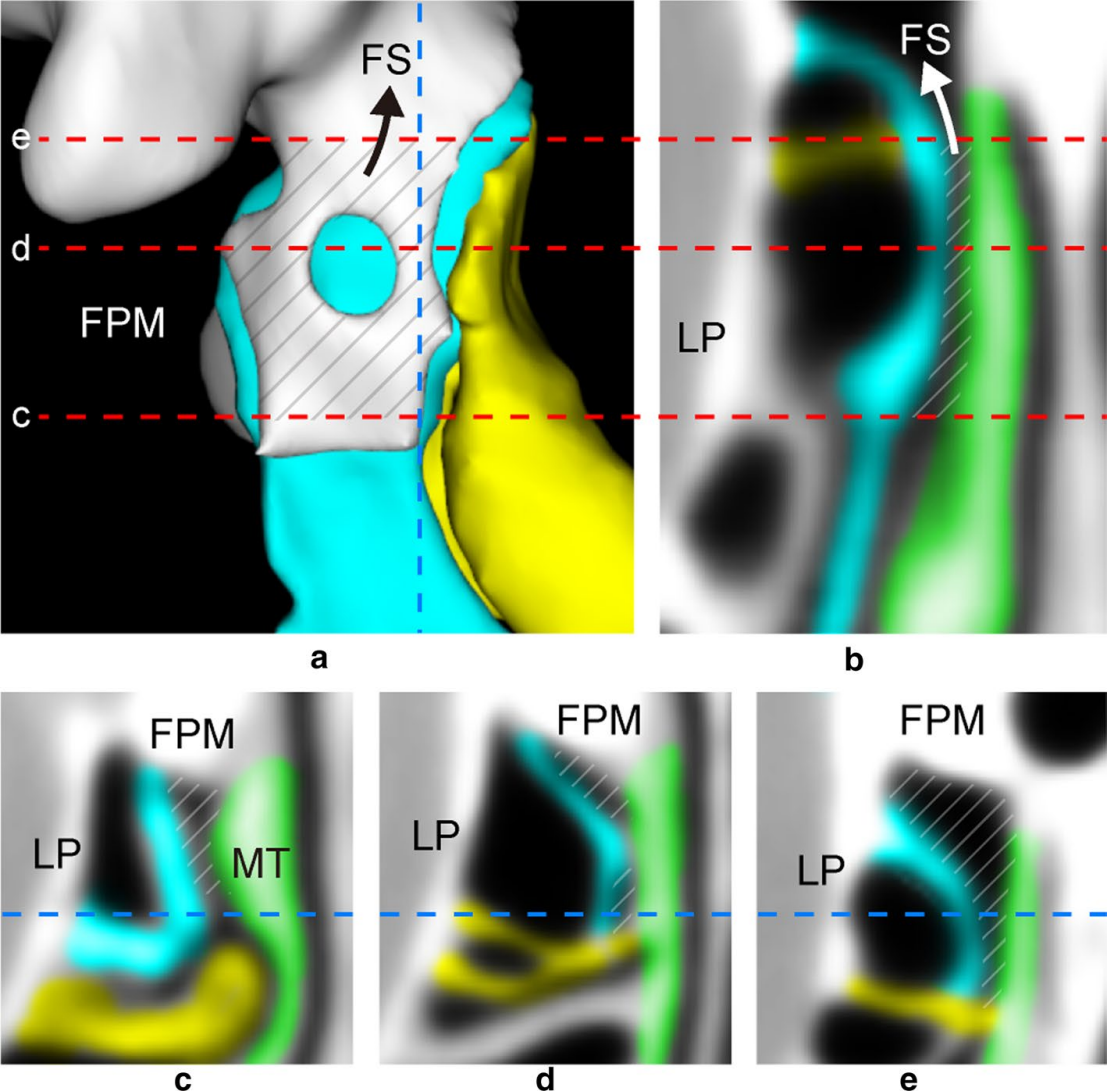
**a** Medial view of 3D reconstruction of [UP–MT- > FS]. (MT removed.) [UP–MT- > FS] arises from [UP–MT] (black arrow). Vertical dotted line indicates coronal slice (**b**). Horizontal dotted lines indicate axial slices (**c**–**e**). **b** Coronal MPR showing transition from [UP–MT] to [UP–MT- > FS] (white arrow). **c**–**e** Axial MPR. blue = uncinate process (UP); yellow = basal lamella of ethmoidal bulla (BLEB); green = middle turbinate (MT); diagonal lines = [UP–MT]; *LP* lamina papyracea, *MT* middle turbinate, *FS* frontal sinus, *FPM* frontal process of maxilla

**Fig. 3:**
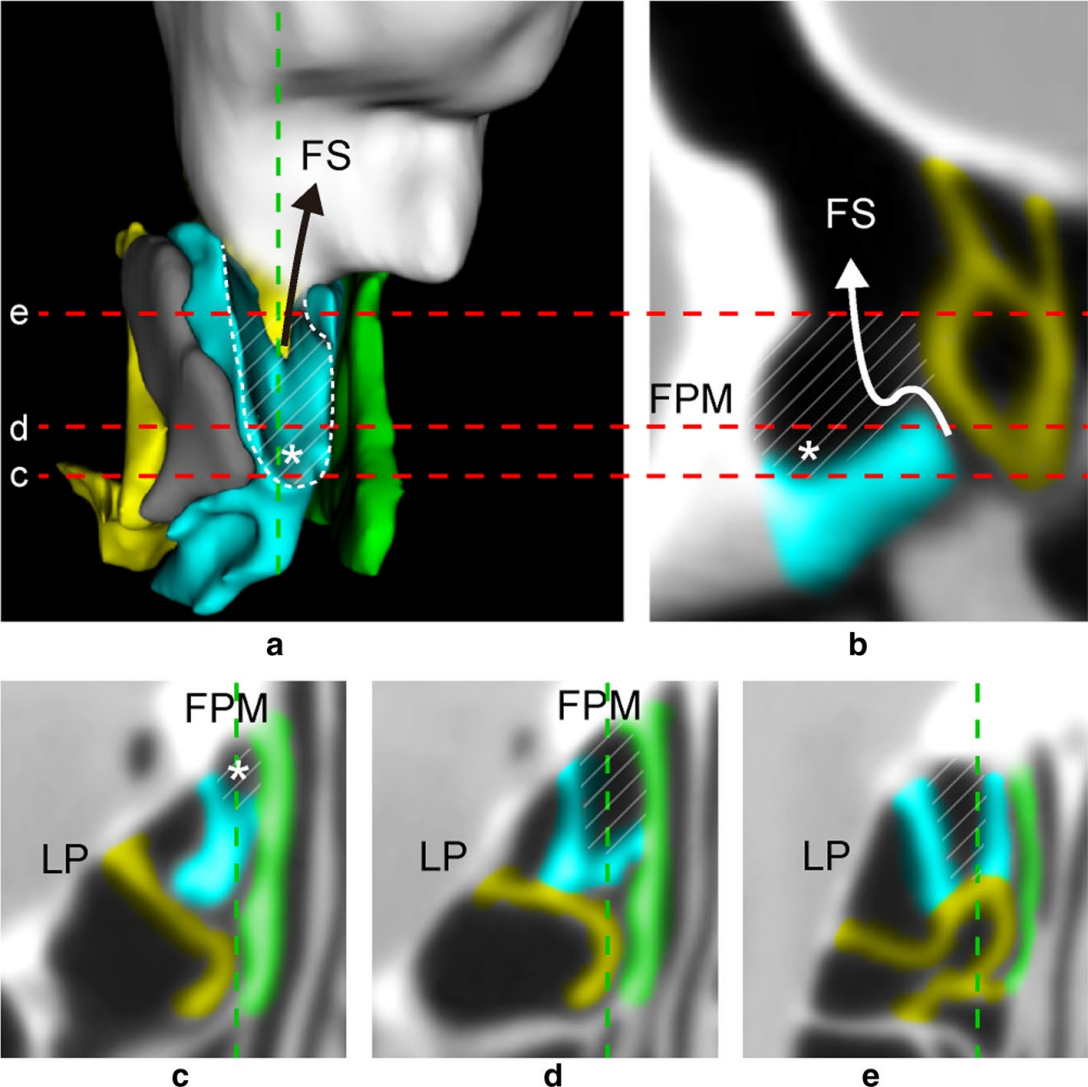
**a** Anterior view of 3D reconstruction of [UP–UPa- > FS] showing [UP–UPa] (white dashed line) with terminal recess (*) at its inferiormost margin. (FPM removed.) Vertical dotted line indicates sagittal slice (**b**). Horizontal dotted lines indicate axial slices (**c**–**e**). Grey indicates [UP–LP- > C]. [UP–UPa] continues to inferior semilunar hiatus (black arrow). **b** Sagittal MPR. [UP–UPa- > FS] joins the inferior semilunar hiatus anterior to BLEB (white arrow). **c**–**e** Axial views of pneumatized-type UPa. **c** [UP–UPa] starts at the inferiormost level of the ethmoid (*). **d** [UP–UPa] joins intersection of [UP–MT] and [BLEB–MT] through inferior semilunar hiatus. **e** [UP–UPa] extends superiorly along the anterior surface of BLEB. blue = uncinate process/accessory uncinate process (UP/UPa); yellow = basal lamella of ethmoidal bulla (BLEB); green = middle turbinate (MT); diagonal lines = [UP–UPa]; *FPM* frontal process of maxilla, *LP* lamina papyracea, *FS* frontal sinus

**Fig. 4:**
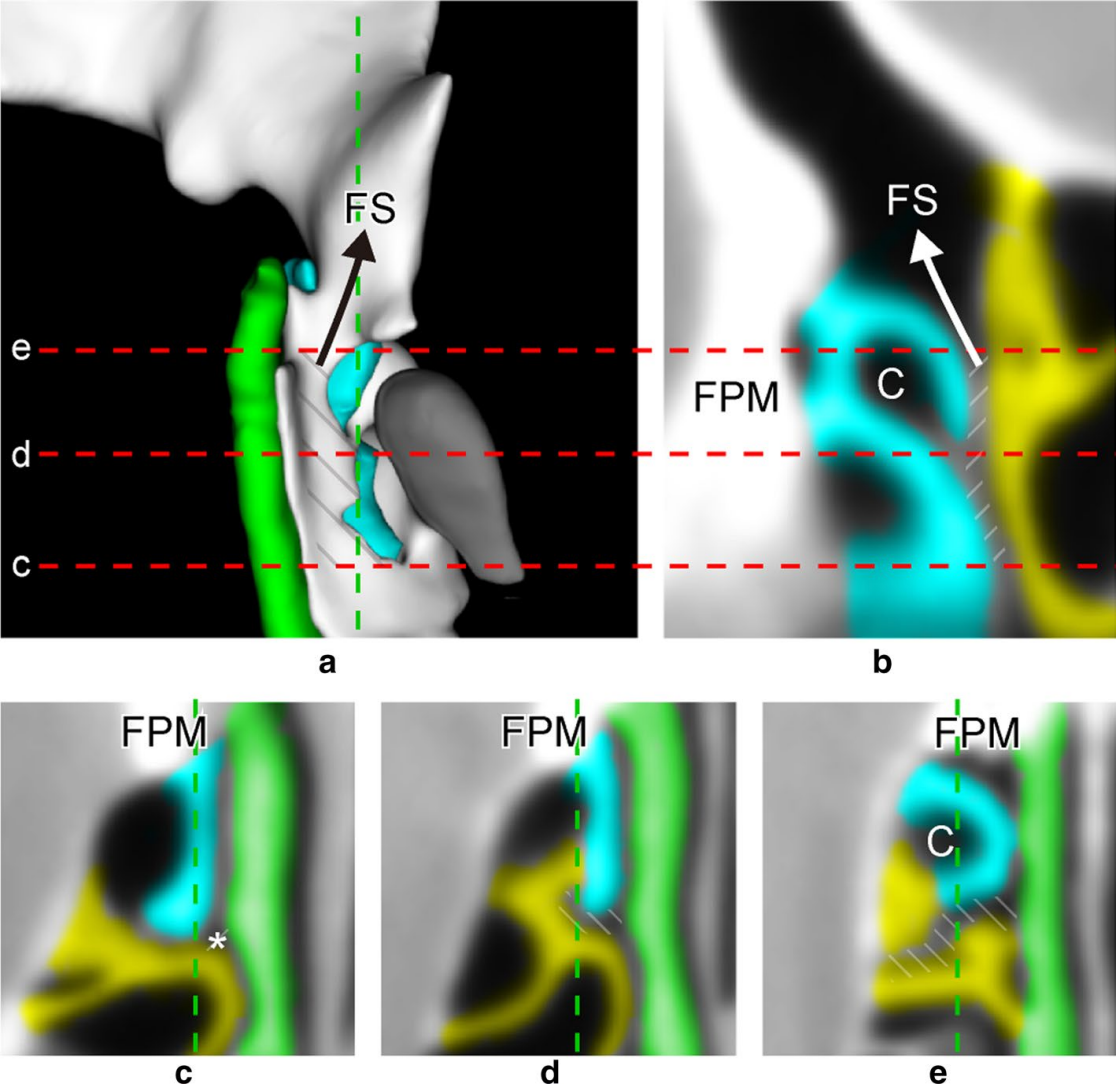
**a** Posterior view of 3D reconstruction showing transition from [UP–BLEB] to [UP–BLEB- > FS] (black arrow). (BLEB removed.) Vertical dotted line indicates sagittal slice (**b**). Horizontal dotted lines indicate level of axial slices (**c**–**e**). **b** Sagittal MPR showing transition from DP to FS (white arrow) and anterior margin of BLEB attaching to skull base, forming the posterior wall of both [UP–BLEB- > C] and [UP–BLEB- > FS]. **c** Axial MPR of [UP–BLEB] starting at intersection of [UP–MT], [UP–LP], and [BLEB–MT] (*). **d** [UP–BLEB] continues medial to juncture of UP and BLEB. In this particular case, at this level, [UP–BLEB] extends anterolaterally to form [UP–BLEB- > C]. **e** [UP–BLEB] forming [UP–BLEB- > C] before becoming [UP–BLEB- > FS]. Note [UP–BLEB] forming a narrow duct, unlike other DPs. blue = uncinate process (UP); yellow = basal lamella of ethmoidal bulla (BLEB); diagonal lines = [UP–BLEB]; *DP* drainage pathway, *FPM* frontal process of maxilla, *FS* frontal sinus

**Fig. 5:**
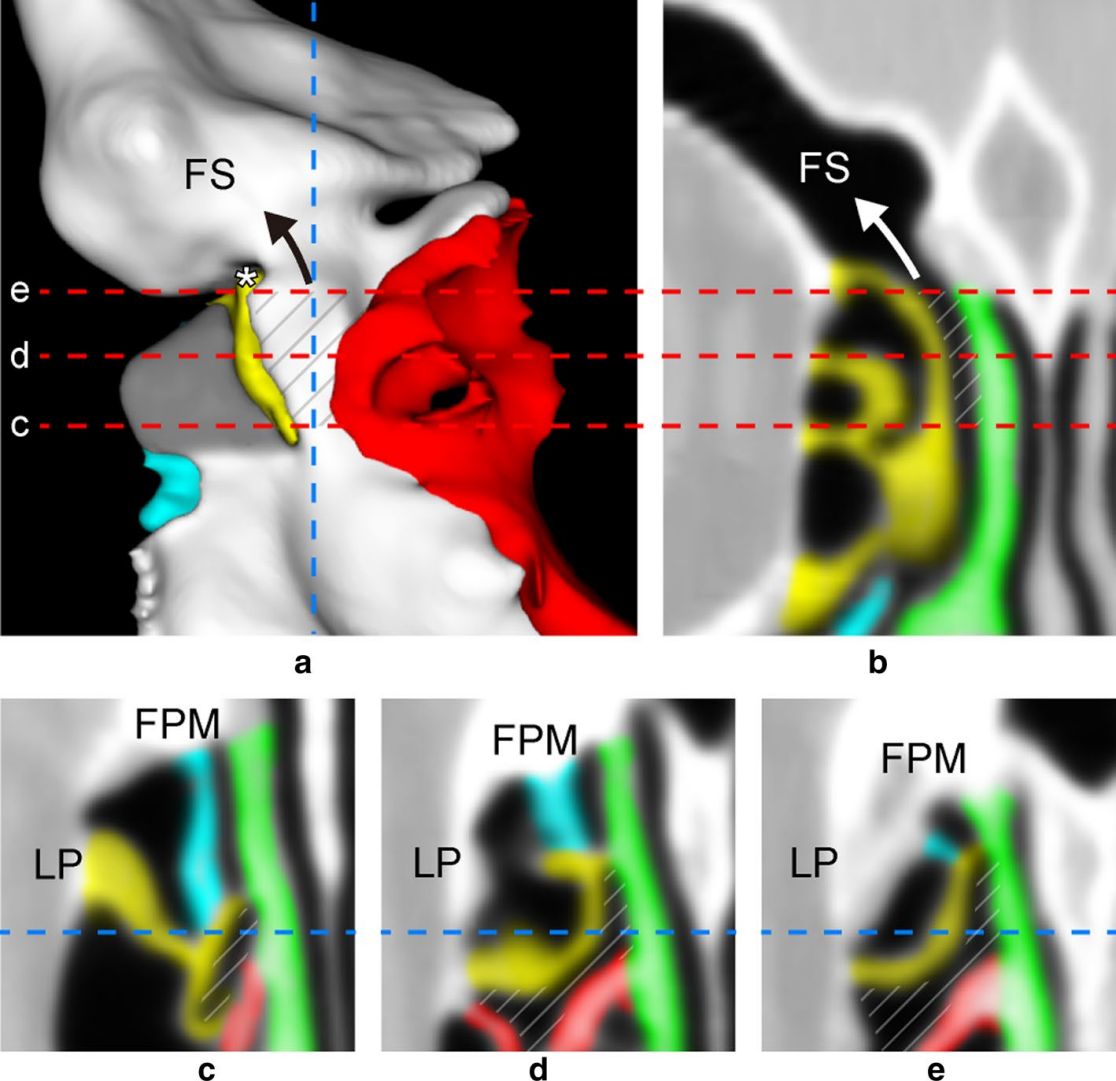
**a** Medial view of 3D reconstruction showing transition from [BLEB–BLMT] to [BLEB–BLMT- > FS] (black arrow). (MT removed.) Dotted lines indicate level of axial slices (**c**–**e**). Note anterior margin of BLEB connects to FPM (*), forming the anterior wall of [BLEB–BLMT- > FS]. Grey indicates [UP–MT]. **b** Coronal MPR showing transition from [BLEB–BLMT] to [BLEB–BLMT- > FS] (white arrow). **c**–**e** Axial MPR showing [BLEB–BLMT] beginning at inferior semilunar hiatus (between BLEB and MT) and then expanding to form [BLEB–BLMT- > FS]. blue = uncinate process (UP); yellow = basal lamella of ethmoidal bulla (BLEB); red = basal lamella of middle turbinate (BLMT); diagonal lines = [BLEB–BLMT], *FS* frontal sinus

1. **[UP**–**LP] between uncinate process and lamina papyracea**
  [UP–LP] was found in 100 sides. [UP–LP- > FS] was found in 11 sides. [UP–LP] occurred in all sides, because it is analogous to the ethmoidal infundibulum (EI). [UP–LP- > C] began at the junction of UP and FPM (the inferiormost agger nasi) and expanded upwards along FPM and LP from EI (Fig. 1A, B). [UP–LP- > C] started from EI and extended along LP. Variations in the size (extent) and shape of [UP–LP- > C] were found. When [UP–LP- > FS] existed, [UP–LP] passed through [UP–LP- > C] without narrowing before reaching EI (Fig. 1C–E).
2. **[UP**–**MT] between uncinate process and middle turbinate**
  [UP–MT] was found in 100 sides. [UP–MT- > FS] was found in 46 sides.
  [UP–MT] began at the inferiormost level of the ethmoid between UP laterally and MT medially, extending superiorly along FPM towards the frontal bone (Fig. 2). [UP–MT] varied and could end in a terminal recess, forming a cell in the frontoethmoidal region (between the ethmoid and frontal sinus), or form FS and a cell or cells within the frontal bone. In this study, up to 2 cells were formed in addition to FS.
3. **[UP**–**UPa] between uncinate process and accessory uncinate process**
  [UP–UPa] was found in 18 sides. [UP–UPa- > FS] was found in 14 sides.
  [UP–UPa] formed between UP and UPa, a lamella attached to FPM roughly parallel to UP. UPa is either a split UP or a pneumatized UP. We give an example of [UP–UPa] created by a pneumatized UP (Fig. 3).
4. **[UP**–**BLEB] between uncinate process and basal lamella of ethmoidal bulla**
  [UP–BLEB] was found in 64 sides. [UP–BLEB- > FS] was found in 24 sides.
  At the inferiormost ethmoid, [UP–BLEB] started at the intersection of [UP–MT], [UP–LP], and [BLEB–MT] (Fig. 4C). At a higher level of the ethmoid, the posterior margin of UP connected to BLEB (Fig. 4D), forming the suprainfundibular plate [7]. [UP–BLEB] ascended the anterior surface of BLEB along the junction with UP, then formed a cell or cells in the anterior ethmoid and FS in the frontal bone (Fig. 4A, , B, E). The position of [UP–BLEB] depended on where UP connected to the anterior surface of BLEB. Unlike the other DPs, [UP–BLEB] has a more variable form, because both UP and BLEB are less predictable than other bony walls. The variable nature of UP and BLEB causes [UP–BLEB] to narrow (Fig. 4D). In this study, if [UP–BLEB- > FS] was present, up to 2 additional cells could exist. In the absence of [UP–BLEB- > FS], there were up to 4 cells anterior to BLEB.
5. **[BLEB**–**BLMT] between basal lamella of ethmoidal bulla and basal lamella of middle turbinate**
  [BLEB–BLMT] was found in 100 sides. [BLEB–BLMT- > FS] was rare, found in only 5 sides (Fig. 5).
  [BLEB–BLMT] began at the superior semilunar hiatus. [BLEB–BLMT- > C] formed posterior to the transition between the ascending and descending crus of BLEB is EB. However, this space may be occupied by a posteriorly extending [UP–LP- > C] or by a cell arising from the superior meatus.

## Discussion

The developmental origins of the drainage pathways of the frontoethmoidal region can help us understand this complex region. Some authors, referencing Terracol and Ardouin, assert that the origin of FS lies in one of three primordial cell groups: orbital, nasal, or bullar [8–10]. When we apply their developmental model to our findings, the orbital cell group seems to correspond to [UP–LP] (ethmoidal infundibulum), the nasal to [UP–MT], and the bullar to [BLEB–BLMT] (superior semilunar hiatus).

The developmental pattern seems to influence the drainage pathway pattern, and the drainage pathway pattern in turn is followed by surgeons during ESS. By following the bony walls of the five drainage pathways, the surgeon can imagine the drainage pathway itself and reach the targeted anterior ethmoid cell or frontal sinus.

From [UP–LP], in the agger nasi, cells form directly from the ethmoidal infundibulum facing FPM anteriorly and LP laterally. The largest [UP–LP- > C] is not confined to the agger nasi but extends superiorly along FPM forming a cell or FS in the frontal bone. [UP–LP- > C] may also expand posteriorly or superiorly along LP forming a large cell that occupies the skull base, suprabullar region, and even the area posterior to BLEB. [UP–LP- > C] dominates the agger nasi, preventing the formation of cells from other drainage pathways.

[UP–MT] medial to UP maintains its shape to the frontoethmoidal region unless [UP–LP- > C] or [UP–BLEB- > C] dislocate UP medially. [UP–MT- > FS] accounted for nearly half (45%) of FS in 100 sides, likely due to the stability of attachment of UP and MT to FPM.

Consistent with Jiang et al., we observed a few cases of UPa anterior to BLEB, distinct from UP and connected to FPM [11]. [UP–UPa] develops superior to the frontal bone between [UP–LP] and [UP–MT].

[UP–BLEB] forms from the anterior surface of BLEB, where the posterior margin of UP connects to BLEB forming the suprainfundibular plate. As noted earlier, unlike the other DPs, [UP–BLEB] tends to form a narrow opening as it is compressed by its lamellae. Pianta et al. describe a singular FSDP dividing the “agger complex” (between FPM and FSDP) and the “bullar complex” (between the FSDP and BLMT), which corresponds roughly to [UP–BLEB] [12]. However, our findings point to 5 possible FSDPs, none of which could be said to divide the anterior ethmoid into compartments.

[BLEB–BLMT] may be referred to as the superior semilunar hiatus. [BLEB–BLMT] forms cells between BLEB and BLMT. The cell formed posterior to the transition between ascending and descending crus of BLEB is called the ethmoidal bulla. In our study, [BLEB–BLMT] could form up to three cells, each from a separate branch, with a multilayered structure posterior to BLEB.

**Fig. 6:**
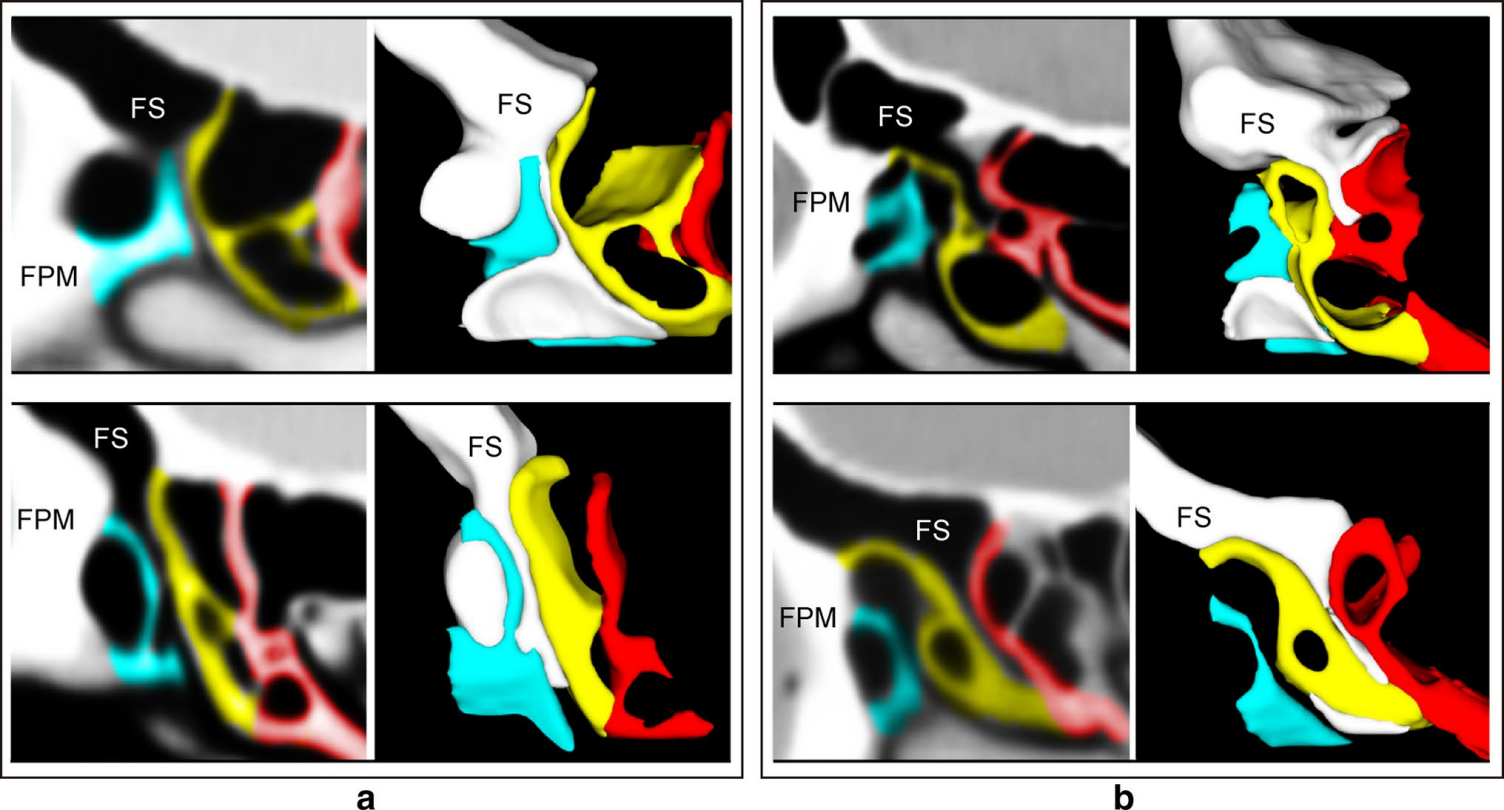
Example of BLEB as posterior wall or anterior wall of drainage pathways. **a** Sagittal MPR and 3D reconstruction (medial to lateral view) showing BLEB as posterior wall of [UP–LP- > FS] (top) and [UP–BLEB- > FS] (bottom). **b** Sagittal MPR and 3D reconstruction (medial to lateral view) showing BLEB as anterior wall of [BLEB–BLMT- > FS]. Note: 3D structures within sagittal MPR have been removed. yellow = basal lamella of ethmoidal bulla (BLEB); blue = uncinate process (UP); red = basal lamella of middle turbinate (BLMT); *FPM* frontal process of maxilla, *FS* frontal sinus

There is a critical difference between [BLEB–BLMT- > FS] and the other four drainage pathways. In [BLEB–BLMT- > FS], the anterior margin of BLEB connects to FPM, with BLEB forming the anterior wall of [BLEB–BLMT]. In contrast, in [UP–LP- > FS], [UP–MT- > FS], [UP–UPa- > FS], and [UP–BLEB- > FS], the anterior margin of BLEB connects to the skull base, forming the *posterior* wall of the drainage pathway (Fig. 6). There are, of course, exceptions, as in Fig. 1B, where [UP–BLEB- > C] forms between [UP–LP- > FS] and BLEB.

In our study, 95% of the FS drainage pathways were anterior to BLEB; in almost all sides, the anterior margin of BLEB connected to the skull base to form the posterior wall of the drainage pathway. Therefore, BLEB, along with UP, may be the most important landmark for approaching the frontal sinus.

We are aware of the limitations of this study. Further study to assess interrater agreement may be necessary to address the possibility of familiarity bias. In addition, the CTs were from healthy subjects whose anatomy was free of bony remodeling. All of the subjects were Japanese; a more heterogeneous study population might reveal different results [13].

We believe, however, that this paper may clarify some issues related to the complex anatomy of the drainage pathways of the frontoethmoidal region: the developmental pathways leads to understanding of the drainage pathways, and the drainage pathways guide the surgeons through the anterior ethmoid to the frontal sinus.

## Conclusions

Beginning in the middle meatus and following the drainage pathways, which themselves follow the developmental pathways, leads us to the cells and sinuses of the frontoethmoidal region. We hope this paper will help other surgeons to perform ESS more safely through a better understanding of the drainage pathways of the frontoethmoidal region.
